# Redistribution of soil water by mature trees towards dry surface soils and uptake by seedlings in a temperate forest

**DOI:** 10.1111/plb.13764

**Published:** 2025-01-17

**Authors:** B. D. Hafner, B. D. Hesse, T. E. E. Grams

**Affiliations:** ^1^ School of Life Sciences, Soil Biophysics and Environmental Systems Technical University of Munich Freising Germany; ^2^ Department of Integrative Biology and Biodiversity Research, Institute of Botany University of Natural Resources and Life Sciences Vienna Austria; ^3^ School of Life Sciences, Land Surface‐Atmosphere Interactions Technical University of Munich Freising Germany

**Keywords:** deuterium labeling, dryland mechanism, hydraulic redistribution, mature mixed beech forest, soil drying–wetting cycles, temperate forest ecosystem, water stable isotopes

## Abstract

- Hydraulic redistribution is considered a crucial dryland mechanism that may be important in temperate environments facing increased soil drying–wetting cycles. We investigated redistribution of soil water from deeper, moist to surface, dry soils in a mature mixed European beech forest and whether redistributed water was used by neighbouring native seedlings.
- In two experiments, we tracked hydraulic redistribution via (1) ^2^H labeling and (2) ^18^O natural abundance. In a throughfall exclusion experiment, ^2^H water was applied to 30–50 cm soil depth around mature beech trees and traced in soils, in coarse and fine roots, and in the rhizosphere. On five additional natural plots, the ^18^O signal was measured in seedlings of European beech, Douglas fir, silver fir, sycamore maple, and Norway spruce at dawn and noon after a rain‐free period.
- We found a significant enrichment in ^2^H in surface soil fine roots of mature beech, and an indication for transfer of this water into their rhizosphere, suggesting hydraulic redistribution from deeper, moist to drier surface soils. On four of the five additional plots, δ^18^O of seedlings' root water was lower at dawn than at noon. This indicated that dawn root water originated from soil layers deeper than the seedlings' rooting depth, suggesting hydraulic redistribution by neighbouring mature trees.
- Hydraulic redistribution equated to about 10% of daily transpiration in mature beech trees, and contributed to root water in understory seedlings, emphasizing hydraulic redistribution as a notable mechanism in temperate forests. Transport mechanisms and potential of different tree species to redistribute water should be further addressed.

## Graphical abstract

Mature beech trees redistributed soil water, equal to ca. 10% of stand transpiration, from deeper moist soils to dry surface soils, where it was taken up by seedlings of different tree species.

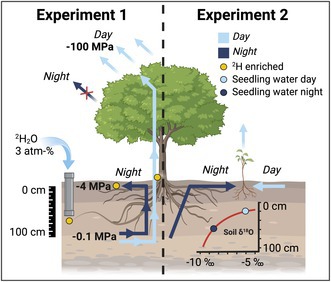

## INTRODUCTION

Hydraulic redistribution is the passive reallocation of water by plants following a gradient from less negative (‘high’) to more negative (‘low’) water potentials (Richards & Caldwell 1987). In the soil, plants with a dimorphic root system can access both surface and deeper soil water (Dawson & Pate 1996; Di *et al*. 2018). Under soil drying conditions, when water in surface soils becomes unavailable for root uptake, deeper soil layers can still contain plant‐available soil water. During nighttime, when plants close their stomata and atmospheric water demand is low, surface soil water potential may be lower than leaf water potential. Hence, water from deeper soils, and even the aboveground tissues, where water potential is higher, can be moved and released to dry surface soils, where the water potential is lowest, via plant roots (Richards & Caldwell 1987). It has been suggested that the released water may benefit the redistributing plants by functioning as a binding agent between roots and soils, maintaining soil root contact, making nutrients available (Wang *et al*. 2009), encouraging microbial activity (Fu *et al*. 2018), and generally improving survival of roots (Bauerle *et al*. 2008; Liu *et al*. 2021). Occurrence and mechanisms of hydraulic redistribution in temperate ecosystems have been described (Meinzer *et al*. 2004; Nadezhdina *et al*. 2009, 2010; Domec *et al*. 2010; Zapater *et al*. 2011), but its role in temperate forests facing severe droughts and its relevance for neighbouring seedlings still remain largely underexplored.

Temperate biomes have been experiencing more frequent and more intense heat and drought events during the last two decades, and climate predictions suggest a further increase of severe weather conditions in the future (IPCC 2014; Schuldt *et al*. 2020; Senf *et al*. 2020). At the same time, more heavy and short rainfall events are recorded, resulting in more frequent drying–wetting cycles in temperate biomes that could promote the formation of water potential gradients in the soil, e.g. after a longer dry period, when plant‐available water can only be found in deeper soil layers, or after a heavy rain event, when the surface soil experiences higher water potentials than deeper soils (Prieto *et al*. 2012). Hence, hydraulic redistribution of water along the resulting soil water potential gradients may become a more common phenomenon in temperate ecosystems (Grünzweig *et al*. 2022).

In comparison to managed agricultural systems that receive irrigation or grasslands that can shift populations quickly, forest ecosystems react more slowly to climatic changes and need to rely on current species composition and their resistance and resilience against biotic and abiotic stressors in a changing environment (Hartmann *et al*. 2018; Schuldt *et al*. 2020; Wessely *et al*. 2024). Among tree species, different hydraulic strategies have evolved to react to environmental changes, with some species showing rapid responses and being considered as more conservative in their stomatal reaction to drought events (“isohydric”), while other species try to maintain functioning similar to non‐stressed conditions for longer times (“anisohydric”) before showing stress‐induced reactions (Meinzer *et al*. 2016; Fu & Meinzer 2018; Ratzmann *et al*. 2019). Two of Central Europe's economically and ecologically most important tree species, *Fagus sylvatica* L. (European beech) and *Picea abies* (L.) H.Karst. (Norway spruce), can be found on rather opposing sides of this reaction spectrum. Subjected to soil drying, spruce follows a more isohydric strategy, closing its stomata early to avoid low xylem water potentials and cavitation, while beech maintains stomatal conductance at higher levels, risking lower minimum xylem water potentials in its tissues (Pretzsch *et al*. 2014; Martínez‐Vilalta & Garcia‐Forner 2017; Magh *et al*. 2019). Partially, the reason for this behaviour can be found belowground, where beech tends to have a higher portion of its root system in deeper soil layers than spruce (Schmid 2002; Schmid & Kazda 2002; Zwetsloot *et al*. 2019), potentially enabling beech trees to take up deeper soil water reserves than spruce (Kahmen *et al*. 2022). This trend has been observed to be even stronger when the two species are growing in mixture with each other, with beech shifting its average rooting depth to even deeper soil layers compared to respective monocultures (Schmid & Kazda 2002; Goisser *et al*. 2016). Access to deeper water reserves by beech roots poses the question, if an overnight uplift of deeper soil water by beech happens in temperate forests under drought, that may aid beech trees to overcome drought periods, e.g. via keeping surface roots hydrated and maintaining access to nutrients in the surface soil (Wang *et al*. 2009). Further, it remains open if redistributed water can potentially be available to spruce roots in surface soils, ameliorating hydrological conditions for spruce in mixture with beech (He *et al*. 2013; Pretzsch *et al*. 2020).

More frequent drying–wetting cycles in temperate forests may cause trees to rely more on deeper soil water, which challenges the adaptive capacities of different species. In particular, increasing droughts may affect the development of seedlings and saplings. Drought‐induced growth decline and mortality in tree seedlings was especially high during the most recent hot and dry years in Central Europe, but also recovery potentials were observed, which may be highly species‐specific (Beloiu *et al*. 2022). Overnight hydraulic redistribution by neighbouring mature trees may make deeper soil water available to seedlings under drought conditions and contribute to their transpiration demand throughout the following day. However, other than evidence from the Mediterranean (Muler *et al*. 2018), drylands (Warren *et al*. 2008), or from pot studies under controlled environments (Hafner *et al*. 2017, 2021), the occurrence, magnitude, and dynamics of water redistribution from mature trees to neighbouring seedlings in temperate regions remain unknown.

Here, we conducted two experiments to determine (i) hydraulic redistribution by mature beech trees, and (ii) to study seedlings' uptake of redistributed water. In a first experiment, we measured hydraulic redistribution in a long‐term throughfall exclusion study, hypothesizing that mature beech trees redistribute soil water along a water potential gradient from deeper towards dry surface soil layers. To investigate if the redistributed water may be used by neighbouring spruce trees, we further hypothesized that water will be released into the soil surrounding surface beech fine roots. We labelled deeper soils surrounding mature beech trees with ^2^H enriched water to trace the origin of the water sampled in trees' surface roots and soils. In a second experiment, we sampled seedlings of different tree species growing in close vicinity to mature trees in five forest plots during morning and afternoon hours, hypothesizing that seedlings sampled in the morning will contain a fraction of redistributed deeper soil water while the water sampled from seedlings in the afternoon will reflect surface soil water origin. We made use of the fact that in temperate environments there is a gradient in soil water isotopic composition (here: δ^18^O) during summer, decreasing from surface to deeper soil (Brinkmann *et al*. 2018). Hence, we expected δ^18^O of seedlings' xylem water to be lower (i.e., containing deeper soil water with lower δ^18^O from hydraulic redistribution by mature trees) in the morning than in the afternoon (i.e., predominantly containing surface soil water with higher δ^18^O).

## MATERIAL AND METHODS

### Site and experimental setup—Experiment 1

To study hydraulic redistribution by mature beech trees, we traced water flow through roots in a long‐term throughfall exclusion site (Kranzberg forest roof ‘KROOF’ experiment) close to Freising in southern Germany (48°25′9.91″N, 11°39′40.13″E) (Grams *et al*. 2021). The soil is a haplic Luvisol originating from Loess over Tertiary sediments with a silty loam texture and a moder type humus layer. On the site, throughfall exclusion roofs withheld summer precipitation from six experimental plots (115–174 m^2^ each). The experimental plots each comprised 3–7 individuals of mature beech (66 ± 2 years) and spruce (86 ± 4 years) trees, respectively (Pretzsch *et al*. 2014). To avoid potential lateral water flow from outside of the plots, tarps were inserted to 1‐m depth around all sides of the plots. Soil moisture was recorded within beech groups on each plot at four soil depths (0–7 cm, 10–30 cm, 30–50 cm, and 50–70 cm) using vertically installed custom built 3‐rod TDR sensors (20 cm length). Soil water retention was estimated from disturbed soil samples taken outside the plots at 10 to 40 cm depth using a combination of hanging column and the pressure plate method (Dane & Hopmans 2002a, 2002b) (see Supplementary data—Data S1). Water content readings were transformed to soil water potentials according to the determined retention curve, and log10 transformed to represent pF values.

One beech tree per plot was selected to assess potential hydraulic redistribution, and 50 PVC plastic tubes (1 m length, 13 mm inner diameter; MCM Systeme, Viersen, Germany) were inserted vertically to a depth of 50 cm in a semicircle around the tree trunk. The depth placement of the tubes followed indication that beech roots were still highly abundant at that depth, while hardly any roots of spruce trees could be found at this depth (Goisser *et al*. 2016; Zwetsloot & Bauerle 2021). The closest tubes were 50 cm away from the trunk and the tubes had a distance of 30 cm from each other (see Fig. S1). The bottom 20 cm of the tubes was perforated with a drill (diameter of holes 4 mm, ca. 2 holes per cm^2^), and the bottom opening was sealed with a plug so that water could only leave through the drilled holes on the sides of the tube. Tubes were inserted 1 year before the experiment to ensure healing of potential root damage due to drilling and tap water was regularly given to the tubes to stimulate root growth around the tubes (once to twice per month over 3 months prior to the experiment, about 100 ml per tube).

In July 2017, the third season of experimental summer drought on the site, a total of 7 L of ^2^H enriched water (ca. 3 atom‐%) was added through the tubes to each of the study trees over the course of 7 days. Seven days before and 7 days after the labeling began, soil cores, coarse and fine root samples, as well as rhizosphere of fine roots were collected at dawn (between 05:00 and 08:00 h). Soil cores were collected close to the labeling tubes (ca. 15 cm distance to the closest tube) with a 1 m core (diameter: 2 cm; Pürkhauer) to a depth of 90–100 cm and subsequently separated into 10 cm depth increments and collected in 12 ml exetainer vials (LabCo, Lampeter, UK). In addition, surface soils were sampled with cork borers (6 mm inner diameter) from the top of the mineral soil layer after carefully removing the organic layer. Surface soil samples were separated into three depth increments (0–3 cm, 3–6 cm, and 6–10 cm soil depth) and stored in 12 ml exetainer vials. Coarse root samples (*n* = 2 per study tree and time point) were collected from the root collar of roots facing towards the labeling tubes, using a cordless electric drill, into another 12 ml exetainer vial. Finally, fine root segments (ca. 15 cm length, 1–3 order roots, *n* = 3 per study tree and time point) were carefully excavated from surface soils between the trunk and the closest labeling tube (ca. 30 cm distance between root and closest tube) by removing the organic soil layer. The rhizosphere soil around the fine roots was collected into 12 ml exetainer vials using spatulas and by carefully shaking the roots. Care was taken that no root tissue was sampled into the rhizosphere vials. After removing the rhizosphere soil from root segments, the fine roots were cut from the tree and sampled in separate 12 ml exetainer vials. All samples were stored at −20°C until further processing.

### Site and experimental setup—Experiment 2

In August 2016, seedlings (ca. 1–3 years old, 10–20 cm height) of silver fir (*Abies alba* MILL., henceforth ‘fir’), sycamore maple (*Acer pseudoplatanus* L., henceforth ‘acer’), beech (*F. sylvatica*), spruce (*P. abies*) and Douglas fir (*Pseudotsuga menziesii* (MIRBEL) FRANCO) were sampled from five forest plots in Thalhauser forest (48°25′05.6″N, 11°41′54.8″ E) and Kranzberger forest (48°24′36.7″N, 11°39′24.5″ E) close to Freising in southern Germany (see Supplementary map—Data S1). Sampled seedlings were in the understory of mature trees, with all but one of the selected plots (plot IV) having beech trees as dominant species in the overstory (Table 3). The five forest plots were selected according to sufficient abundance of seedlings (i.e. >10 seedlings of the same size (Table 3) per species) and a comparable mature overstory density (judged by eye).

Sampling started after a 3‐week rainless period, indicating that a gradient in soil water potential between deep and surface soil had established, during warm weather conditions (25.0 ± 0.6°C day, and 20.2 ± 1.6°C night). In each of the plots, five seedlings per study species (Table 3) were each sampled at dawn (05:00–07:00 h) and in the afternoon (13:00–17:00 h) of the consecutive day. Seedlings were carefully and quickly excavated, freed from rhizosphere soil, the root length was assessed as a proxy of maximum rooting depth, and the root was sampled into 12 ml exetainer vials. Additional soil cores (Pürkhauer, diameter 2 cm; *n* = 3 per plot) were taken to a depth of 80 cm, and the soil sampled into 12 ml exetainer vials divided in four depth increments (0–10 cm, 10–30 cm, 30–60 cm, and 60–80 cm). All samples were stored at −20°C until further processing.

### Water extraction and isotope analysis

Water was extracted from soils and plant tissues via cryogenic vacuum extraction for 120 min (West *et al*. 2006). Subsequently, samples were analysed for ^2^H (Experiment 1) and ^18^O (Experiment 2) signatures with an isotope ratio mass spectrometer coupled to a multiflow system (Isoprime, Elementar, Hanau, Germany). Samples were corrected with two in‐house monitoring standards (heavy: δ^2^H = 133.33‰ and δ^18^O = 13.84‰; light: δ^2^H = −159.41‰ and δ^18^O = −21.58‰) and are expressed in the delta notation against the VSMOW2 standard.

### Mixing models and statistical analyses

We used isotope mixing models to estimate the fraction of labelled water redistributed by coarse roots in fine roots ($f_{\text{fine}-\text{roots}}$) and the rhizosphere ($f_{\text{rhizo}}$) in Experiment 1.

(equation 1) ffine−roots=δHfine−rootst72−δHfine−rootst−72δHcoarse−rootst72−δHfine−rootst−72

(equation 2) frhizo=δHrhizot72−δHrhizot−72δHcoarse−rootst72−δHrhizot−72

We calculated that the isotopic composition of the water of surface fine roots 7 days after labeling (δHfine−rootst72) was a mixture of the isotopic composition of the water in surface fine roots before labeling (δHfine−rootst−72) and the isotopic composition of the water in the coarse roots 7 days after labeling (δHcoarse−rootst72; Equation 1). Similarly, we assumed that the isotopic composition of the water in the rhizosphere of surface fine roots 7 days after labeling (δHrhizot72) was a mixture of the isotopic composition of the water in the rhizosphere of surface fine roots before labeling (δHrhizot−72) and the isotopic composition of the water in the coarse roots 7 days after labeling (δHcoarse−rootst72; Equation 2).

Statistical analysis was done in R in the RStudio environment (R Development Core Team 2023). For each experiment, isotope values were checked for significant differences before and after the labeling (Experiment 1) and between dawn and afternoon (Experiment 2) using linear mixed effect models (‘lme’ function in the ‘NLME’ package; v.3.1–137, Pinheiro *et al*. 2018). Specifically, in Experiment 1, isotope values were tested against the fixed variables time (before or after labeling) and tissue (soil, coarse root, fine root, rhizosphere), with the plot‐ID as a random factor. In Experiment 2, isotope values were tested against time (dawn, afternoon), tissue (soil, root), and sapling tree species, with the forest plot as random factor. Models were checked for normal distribution of residuals (Shapiro test) and variance homogeneity (‘LeveneTest’ function in the ‘*CAR*’ package, v.2.1–2; Fox & Weisberg 2019) and values were transformed to meet test requirements if necessary. Statistical differences were post‐hoc calculated with the emmeans function (package ‘*EMMEANS*’ v.1.5.2–1; Searle *et al*. 1980). In the case of highly enriched samples in soils and coarse roots in experiment 1, transformation was not possible and a Kruskal‐Wallis rank sum test (Package ‘*stats*’ v. 4.3.1) with Dunn post‐hoc test using the Benjamini‐Hochberg method (package ‘*dunn.test*’ v. 1.3.5; Dunn 1964) was used to determine significant differences. Values are given as meas ±1 standard error (SE).

## RESULTS

### Experiment 1

#### Soil water content and water potential

Volumetric soil water content (SWC) was measured before the two sampling timepoints (7 days before and 7 days after labeling). There was no significant difference between the time points, however from deeper to surface soil, SWC decreased significantly, ranging from 28.7 ± 1.0 vol‐% in the deepest to 8.3 ± 0.6 vol‐% in the surface soil (Table 1). This corresponded to soil water potentials (SWP) and pF values close to field capacity in the deeper soil layers (−0.04 ± 0.01 MPa or a pF of 2.5 ± 0.1 in 30–50 cm and −0.01 ± 0.00 MPa or a pF of 2.1 ± 0.1 in 50–70 cm), while in the surface soil, SWP was below −1.5 MPa (pF of 4.2), generally considered as the plant wilting point (−4.46 ± 1.60 MPa or a pF of 4.4 ± 0.1 in 0–7 cm; Table 1), indicating a strong gradient in soil water potential between the moist labelled deeper (30–50 cm) and the dry surface soil (0–7 cm), where fine roots were sampled. The SWC at the exact labeling spots may have been even a little higher due to the addition of 7 L of ^2^H enriched water, further increasing the moisture gradient between deeper and surface soils. Using measured thresholds for water availability on the plots (Grams *et al*. 2021), we calculated transpirable SWC in the experimental plots (Klein *et al*. 2014). Across soil depths, we estimated 19.2 mm of transpirable SWC, however, the surface soil only contributed 0.6 mm, confirming low water availability to roots in the surface soils (for details see Supplementary data—Data S1).

**Table 1:** Soil water content, soil water potential and pF values AVERAGED from two timepoints each 7 days before and 7 days after the ^2^H_2_O labeling event. Values were measured in 4 different soil depths on 6 experimental plots.

depth	SWC (vol‐%)	SWP (MPa)	pF
0–7 cm″ S″	8.3 ± 0.6^a^	−4.46 ± 1.60^a^	4.4 ± 0.1^a^
10–30 cm	16.5 ± 0.9^b^	−0.18 ± 0.04^b^	3.1 ± 0.1^b^
30–50 cm″ L″	22.5 ± 1.1^c^	−0.04 ± 0.01^c^	2.5 ± 0.1^c^
50–70 cm	28.7 ± 1.0^d^	−0.01 ± 0.00^d^	2.1 ± 0.1^d^

Small letters indicate significant differences between soil depths. Capital letters indicate labeling (“L”) and fine root sampling depth (“S”).

#### Soil and plant isotopes

**Fig. 1:**
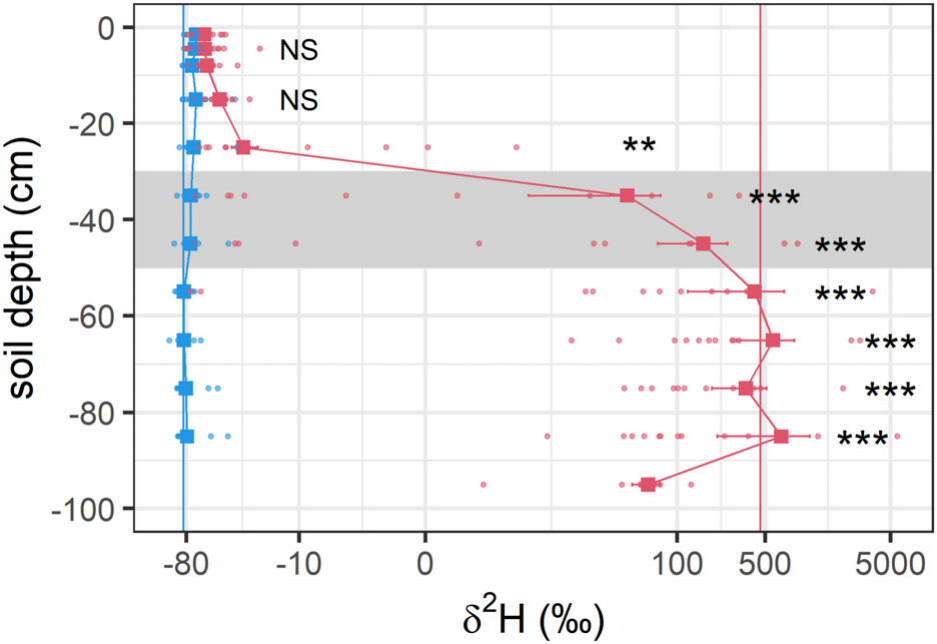
^2^H signatures in the soil 7 days before (blue dots and line) and 7 days after (red dots and line) the labeling started. The shaded area indicates the labeling depth (30–50 cm), and vertical lines indicate coarse root δ^2^H for reference before (blue) and at the end of the labeling (red). Asterisks give significant differences before and after labeling (** < 0.01, *** < 0.001, NS = not significant). Note that no soil in 100‐cm depth was taken prior to the labeling and the *x*‐axis is log‐scale.

Seven days after the labeling, the soil was significantly enriched in ^2^H at the labeling depth (30–50 cm) and below (until ~100 cm soil depth). Above the labeling depth, we only found an enrichment in the next layer (20–30 cm: from −70 ± 4‰ to −28 ± 7‰; *p* = 0.01), whereas the bulk‐soil above was not enriched in ^2^H (Fig. 1).

Prior to labeling, coarse roots had an average δ^2^H of −84 ± 2‰, intercepting with soil water δ^2^H in 50–70 cm soil depth (Table 2, Fig. 1). Fine roots and their rhizosphere in the surface soil had significantly higher ^2^H signatures of −55 ± 3‰ and − 62 ± 2‰, respectively (Table 2). After the labeling, coarse roots were significantly enriched in ^2^H with 461 ± 136‰ on average. The ^2^H signatures again reflected well on the soil ^2^H signatures in 50–90 cm soil depth (Table 2, Fig. 1). Indicating HR of soil water from below 30 cm soil depth, the water in the fine roots in the surface soils was also significantly enriched by about 10‰ δ^2^H, with an average across all plots of −45 ± 4‰, while the rhizosphere soil by trend (*p* = 0.07) showed higher δ^2^H with −50 ± 6‰, average (Table 2).

**Table 2:** ^2^H signatures in coarse roots, fine roots, and their rhizosphere soil 7 days before and 7 days after the labeling started as well as fraction of labelled water found in fine roots and their rhizosphere 7 days after the labeling (Equations 1 and 2).

content	δ^2^H before labeling (‰)	δ^2^H after labeling (‰)	fraction of labelled water after labeling (%)
Coarse Root	−84 ± 2^a^	461 ± 136***^,a^	
Fine Root	−55 ± 3^b^	−45 ± 4*^,b^	3.7 ± 2.4
Rhizosphere	−62 ± 2^b^	−50 ± 6°^,b^	4.9 ± 3.2

Symbols after labeling indicate significant differences in δ^2^H to before labeling (*** < 0.001, * < 0.05, ° = 0.07) and letters indicate significant differences between sampled tissues before or after labeling.

#### Fraction of labelled water in fine roots and rhizosphere in the surface soil

On day 7 after the labeling, we found a range from 0% to 31% of water in fine roots in the surface soil originating from redistribution by the coarse roots. On average 3.7 ± 2.4% of the water found in fine roots was redistributed water (Table 2). Albeit only enriched by trend (Table 2), we calculated the contribution of redistributed water from coarse roots also in the rhizosphere and estimated an average of 4.9 ± 3.2% of redistributed water on day 7 after the labeling (Table 2).

### Experiment 2

#### Soil water isotopes

**Fig. 2:**
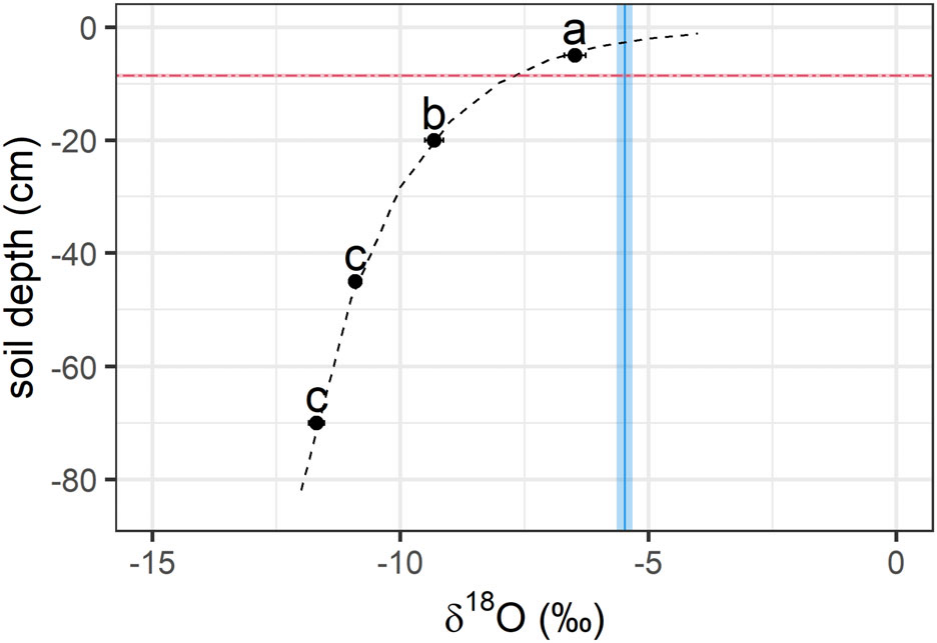
δ^18^O of soil water across soil depths averaged across sampling at dawn and afternoon (average over 5 plots). Different letters indicate significant differences between soil depths, the dashed black line shows an exponential trend line, the dashed red line indicates average seedling root length/maximum rooting depth and the solid blue line average seedling δ^18^O for reference (both ±1SE indicated by the shaded area).

We did not find a difference in soil water isotopic composition between dawn and afternoon (*p* = 0.54) on any of the sampling plots in Experiment 2. Only depth had a significant influence on the δ^18^O (*p* < 0.001) of the soil water. The δ^18^O became more negative with increasing depth, providing a clear and distinct signal between surface (uppermost 10 cm) and deeper soil water (below 20 cm; Fig. 2).

#### δ ^18^O in seedlings at dawn and in the afternoon

**Fig. 3:**
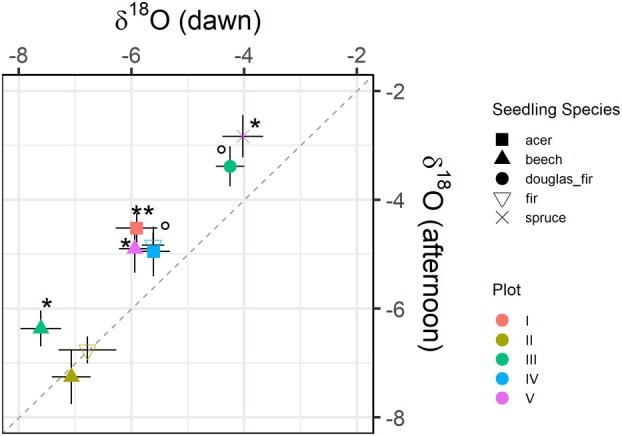
δ^18^O of seedlings' root water sampled at dawn (05:00–07:00 h, *x*‐axis) and in the afternoon (13:00–17:00 h, *y*‐axis). The dashed line indicates the 1:1 line. Different symbols indicate the 5 different seedling species and different colours show the 5 plots that were sampled. Other than species on plot 2 (ocher symbols), all samples are on the left side of the 1:1 line, i.e., showing lower δ^18^O at dawn than in the afternoon. Asterisks indicate significant differences between dawn and afternoon sampling (** < 0.01, * < 0.05, ° ≤ 0.1).

The average water δ^18^O in seedling roots was −5.5 ± 0.2‰ and therefore higher than soil water δ^18^O at the maximum rooting depth of the seedlings (compare intersection of red and blue lines with dashed black line in Fig. 2), suggesting an average water uptake depth of around 3.4 cm. At dawn, the average δ^18^O in seedlings' root water was −5.9 ± 0.2‰ and significantly lower than in the afternoon at −5.1 ± 0.2‰ (*p* < 0.01). Seedlings of all analysed species showed decreased ^18^O water signatures at dawn compared to afternoon, apart from one plot (plot II), where we did not detect differences between dawn and afternoon, irrespective of the seedling species (Fig. 3).

#### Seedling root length and correlation with δ ^18^O

Root length varied significantly between seedling species (Table 3). The longest roots were 13.2 ± 1.1 cm and the shortest 3.5 ± 0.4 cm (Table 3). On average, roots had a length of 8.6 ± 0.3 cm (Fig. 2). There were no differences in root length between sampling times within the sampled species per plot (Table 3).

**Table 3:** Root length of sampled seedlings in experiment 2 on five plots (dominant species in the canopy are given) at dawn and in the afternoon.

plot	dominant canopy species	sampled seedlings	root length (cm) dawn	root length (cm) afternoon
I	Beech	Acer	9.6 ± 1.2^abc^	8.2 ± 0.4^abc^
II	Beech	Beech	10.0 ± 0.9^abc^	9.7 ± 0.9^ac^
II	Beech	Fir	11.1 ± 1.8^ac^	9.5 ± 0.4^ac^
III	Beech/Douglas fir	Beech	13.2 ± 1.1^a^	11.1 ± 0.6^ac^
III	Beech/Douglas fir	Douglas fir	4.3 ± 0.2^b^	3.5 ± 0.4^b^
IV	Fir	Acer	8.4 ± 0.6^abc^	8.4 ± 0.7^abc^
IV	Fir	Fir	6.6 ± 0.5^bc^	6.1 ± 0.7^abc^
V	Beech/Larch	Beech	12.1 ± 0.4^a^	11.2 ± 0.4^a^
V	Beech/Larch	Spruce	5.5 ± 0.7^bc^	5.6 ± 0.7^bc^

Letters indicate significant differences between seedlings at the respective sampling times. There were no differences in root length for any sampled seedlings between dawn and afternoon.

**Fig. 4:**
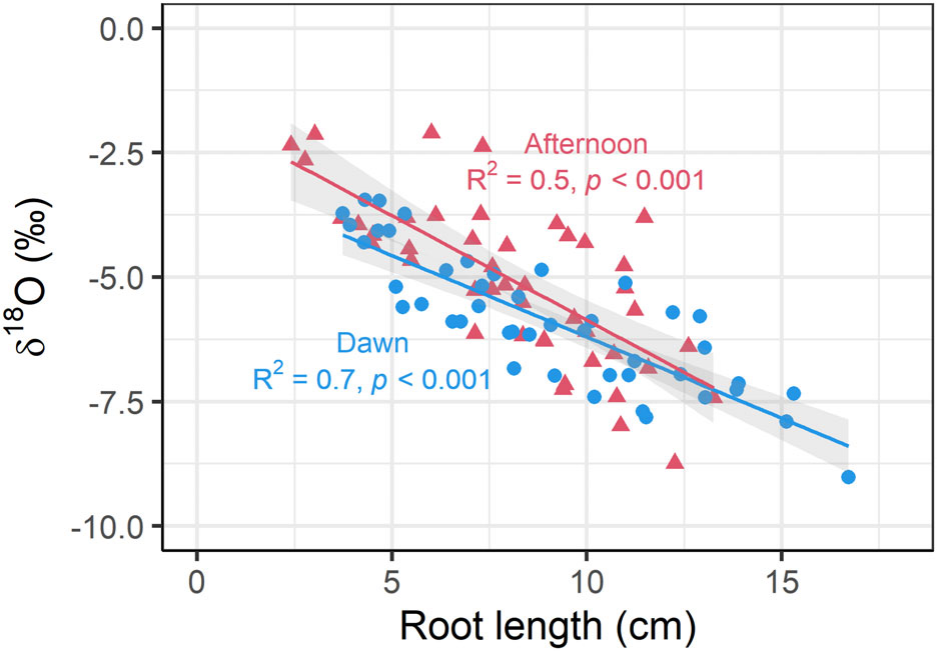
Correlation of root length and δ^18^O of seedlings' root water in Experiment 2. Root water ^18^O signature significantly decreased with increasing root length, irrespective of sampling time. Differences in δ^18^O between dawn (blue dots and regression line) and afternoon (red triangles and regression line) were about 1‰ on average.

Root length was significantly correlated with root δ^18^O water (dawn: R^2^ = 0.7, *p* < 0.001, afternoon: R^2^ = 0.5, *p* < 0.001). Per 1 cm increase in root length, the δ^18^O decreased by ca. 0.4‰, reflecting the decrease in water ^18^O signature with soil depth (Figs. 2, 4). The bulk of root lengths were similar between dawn and afternoon, suggesting negligible influence on the observed difference in δ^18^O between the two time points (Fig. 4).

## DISCUSSION

We investigated hydraulic redistribution of deep soil water in temperate forests. In accordance with our hypothesis, we found that mature beech trees took up deeper soil water (50–70 cm depth) and redistributed a portion of it to their roots and rhizosphere in the dry surface soil, where it may have been available to neighbouring spruce trees. Our second experiment suggests that neighbouring seedlings took up redistributed water at dawn and used this water source by the afternoon.

### Redistribution of deep soil water by mature beech

The ^2^H signature in the coarse root water, both before and at the end of the labeling interval, reflected the isotopic signature in the soil water at 50 cm depth and below, where soil water was generally plant‐available (SWP close to the field capacity), indicating root water uptake depth of the sampled coarse roots at these soil depths (Kahmen *et al*. 2022).The enrichment in the coarse roots at the end of the labeling interval confirmed uptake of the applied ^2^H tracer by the trees to a large extent, since the δ^2^H reflected values in the soil at the labeling depth. The fine roots of beech trees in the surface soils were significantly enriched at the end of the labeling period, and our mixing model revealed that about 4% of the water found in the fine roots originated from hydraulic redistribution. This reflects very well values in beech saplings from greenhouse studies (Hafner *et al*. 2017) and is in the range of other studies using stable isotope labeling approaches at drier sites (Prieto & Ryel 2014). Here, we also found a trend of water flow into the rhizosphere of beech, where the water originating from coarse roots may have contributed to about 5% of the total water amount.

We did not find any significant enrichment of our tracer in the bulk soil above a soil depth of 20 cm (Fig. 1), indicating that the tracer was not transported e.g. via capillary rise within the soil during the measurement period. However, in contrast to results found in oak trees (Zapater *et al*. 2011), we cannot confirm that redistributed water was moved from surface roots into the bulk soil, at least not in amounts high enough to be detected by our approach. Therefore, redistributed water might have only been available to neighbouring spruce trees if their roots directly grew into the rhizosphere of beech roots, or if they were linked through e.g. a common mycorrhizal network (Egerton‐Warburton *et al*. 2007; Querejeta *et al*. 2003).

### Scaling to plot level

For a rough estimation of the total amount of hydraulically redistributed water and its contribution to the water balance of the forest, we weighed the exetainer vials containing root samples pre‐ and post‐water extraction to assess total root water (average of 0.4 ± 0.1 g; not shown) and estimated sampled root dry weight (average of 0.6 ± 0.1 g; not shown). Multiplying by the average fraction of redistributed water in the fine roots (3.7 ± 2.4%), roots contained 0.05 ± 0.03 ml g^−1^ redistributed water per root dry weight. Dry fine root biomass in the surface soils (0–7 cm) was at 1,539.5 ± 172.8 g m^−2^ (analysis of unpublished data from Weikl *et al*. 2023). Therefore, if all fine roots in the surface soil received redistributed water equally, 107.6 ± 61.9 ml m^−2^ (or 0.1 mm) of water was redistributed from coarse roots to fine roots in the dry surface soils in total, which is in the range of other empirically determined values across biomes (Neumann & Cardon 2012). Beech trees transpired, on average, 29 L of water per day (Hesse *et al*. 2024), and had a tree density of 325 stems ha^−1^ (Pretzsch *et al*. 2014), resulting in approximately 1000 mL m^−2^ (or 1 mm) of transpiration per day. Hence, hydraulically lifted water would account for roughly 10% of daily transpiration, which is in line but towards the lower end of previously reported contributions across biomes (Brooks *et al*. 2002; Neumann & Cardon 2012), and 17% of transpirable SWC (0.6 mm) in the surface soil. Spruce trees only used 9 L of water per day (Hesse *et al*. 2024) and at a stand density of 302 stems ha^−1^ (Pretzsch *et al*. 2014), less than 300 ml m^−2^ (or 0.3 mm) was transpired each day. Therefore, even if only a minor fraction of redistributed water from beech reached spruce trees, the relevance may be substantial.

### Seedling uptake of hydraulic redistribution water

The ^18^O signature in seedlings root tissue water at dawn was lower than in the afternoon in four out of five sampled plots (Fig. 3). This suggests water uptake by seedlings from deeper soil layers at dawn compared to the afternoon, as soil water δ^18^O decreased with soil depth (Fig. 2). We did not see a significant difference in rooting depth between seedlings sampled at dawn in comparison to seedlings sampled in the afternoon (Table 3; Fig. 4). Furthermore, isotopic composition of soil water did not change during the day. To reflect seedlings' root δ^18^O at dawn, water uptake depth would have to be shifted by about 2 cm (from ca. 3.5 cm to 5.5 cm; Fig. 2), which, however, would exceed the maximum root length of a couple of sampled seedlings (Table 3, Fig. 4). Additionally, it seems rather unlikely that, at dawn, when temperatures were lower and humidity was higher, water uptake depth was deeper than in the afternoon, when atmospheric vapour pressure deficit was highest. Thus, we conclude δ^18^O root tissue water in seedlings at dawn was a mixture of surface and deep soil water redistributed from neighbouring mature trees. This results in reduced δ^18^O in seedlings' roots at dawn compared to the afternoon, with no diurnal changes in average root water uptake depth.

The difference between dawn and afternoon in δ^18^O of seedings' root water was independent of species of either seedling or overstory tree. There was no preferential flow between mature trees and seedlings from the same species, and mixed species combinations showed the same pattern, similar to previous findings (Hafner *et al*. 2021). It is known that some tree species are colonized by the same mycorrhizal species, but here we even found a potential water redistribution between species colonized by ectomycorrhizal fungi (beech and fir) and arbuscular mycorrhizal fungi (*Acer*), which usually do not form a common network (Lang *et al*. 2011; Kubisch *et al*. 2015). Additionally, also small seedlings showed differences between dawn and afternoon (Fig. 4) while the extension of their associated mycorrhizae was likely significantly lower than in bigger seedlings. This questions the mycorrhizal transport path and raises the question of how the water was transported from the rhizosphere of the redistributing trees to the seedlings. While most plots had beech as dominant overstory species, on one plot (plot IV) fir dominated the canopy. We did not find any difference in redistribution of water from either dominant species, suggesting a similar capacity to redistribute water between the two species.

### Relevance of hydraulic redistribution to seedlings

We roughly calculated the water amounts that were redistributed to obtain an estimation of the importance for seedlings. Assuming a water source of hydraulically lifted water from 50–0 cm depth (as found in beech trees in Experiment 1; δ^18^O of about −11‰; Fig. 2), a mixing model calculation similar to Equations 1 and 2 resulted in a contribution of this deep soil water to water found in the seedlings' roots at dawn of about 16% (see Supplementary information—Data S1). On average, roots contained 0.3 ml of water (determined via weighing of exetainer vials before and after water extraction; not shown), which indicates that 0.05 ml of water found in the roots at dawn were hydraulically redistributed water.

We did not measure soil moisture on the experimental plots or seedling transpiration, but the experiment was conducted under warm conditions after a 3‐week rain‐free period. We cannot conclude the severity of drought on seedlings, but it has been shown that hydraulic redistribution also occurs under moderate drought (Hafner *et al*. 2017). With stronger gradients in soil moisture (Hafner *et al*. 2020), e.g., after a longer drying period, the amount of redistributed water used by the seedlings may be up to or higher than one third of transpired water (Hafner *et al*. 2021), marking the effect as potentially highly relevant for seedlings' water budget under predicted future climatic conditions.

### Conclusions and synthesis

Hydraulic redistribution contributes to the water budget in temperate forests in mature trees and seedlings to a notable amount. Previously mostly studied and quantified as a crucial dryland mechanism, hydraulic redistribution may become more relevant in temperate ecosystems, when moisture gradients in the soil increase under predicted more frequent drying–wetting cycles. The mycorrhizal pathway has been suggested as a major water exchange mechanism (Querejeta *et al*. 2003; Egerton‐Warburton *et al*. 2007; Plamboeck *et al*. 2007; Alagele *et al*. 2021). On one of our plots (plot II; Fig. 3), we did not find a difference in δ^18^O in seedling water between dawn and afternoon. The only difference in this plot compared to the others was that a recent harvest event cleared some of the neighbouring overstory trees and stems were stored for transport close by, potentially disturbing mycorrhizal connections between mature trees and seedlings. However, we also found evidence for water transport between species of different mycorrhization types—although we did not determine mycorrhization and it has been reported that endomycorrhizal trees may host ectomycorrhiza when growing in a mixed host plot (Avital *et al*. 2022; Rog *et al*. 2022). We therefore encourage future studies to investigate the mechanisms of water redistribution between trees, including mycorrhizal colonization and type (Querejeta *et al*. 2012), tree species root morphological characteristics (Sallo *et al*. 2020), tree species identity (Hafner *et al*. 2020), or soil and rhizosphere physical and chemical properties (Wang *et al*. 2009; Montaldo & Oren 2022) on the amount of water that can be redistributed between neighbouring plants. Combined, our Experiments 1 and 2 suggest a transfer of redistributed water from deep‐rooting trees to neighbouring seedlings. However, since we could not measure water transfer from labelled mature beech trees to neighbouring spruce and there were no seedlings on the experimental plots of Experiment 1, direct evidence of water redistribution from mature trees to seedlings should be assessed in future studies.

## FUNDING

BDHa and BDHe were founded by a doctoral scholarship from the German Federal Environmental Foundation (DBU). Support by the German Research Foundation (DFG), the Bavarian State Ministries of the Environment and Consumer Protection as well as Food, Agriculture and Forestry, by the Georg‐Ludwig‐Hartig‐Stiftung and the Europamöbel Umweltstiftung is highly appreciated. The research site is owned by Bayerische Staatsforsten who gave us free access and allowed the experimental setup.

## CONFLICT OF INTEREST

The authors declare no conflicts of interest.

## AUTHOR CONTRIBUTION

BDHa and TG designed the study, BDHa and BDHe collected and analyzed the data. BDHa first wrote the manuscript, and all authors revised and approved the final version of the article.

## Supporting information


**Data S1.** Supporting Information.
