# Supplementary material for: Redistribution of soil water by mature trees towards dry surface soils and uptake by seedlings in a temperate forest
Source: Plant Biol (Stuttg). 2025 Jan 17;28(3):888–96. doi: 10.1111/plb.13764 (PMC13089604; doi:10.1111/plb.13764)
Supplement: Supplementary file 1 — Data S1. Supporting Information. [file PLB-28-888-s001.docx]

Figure 1: Scheme of the experimental setup of experiment 1. A total of 50 tubes were inserted to a depth of 50 cm in a semicircle around a mature beech (Fagus sylvatica L.) tree. Tubes were perforated in the lowest 20 cm with 4 mm drill holes and the bottom was plugged, so that added water could only penetrate the soil from the sides. The closest tubes had 50 cm distance to the tree and the distance between tubes was at least 30 cm. Soil cores were taken to a depth of 90 (100) cm between tubes and fine roots (1-3^rd^ order, about 15 cm length) of the respective beech trees in the surface soil were sampled between the tubes and the tree.

30 cm

50 cm

50 cm

50 cm

20 cm

*Supplementary methods 1; determination of soil water retention curve*

Sampled disturbed soils were dried and sieved (2 mm) and filled into eight 100 cm^3^ stainless steel cylinders to a bulk density of 1.1 g cm^-3^. Cylinders were saturated in de-ionized water, weighed and each four were measured using the hanging column method (water potentials of -3, -10, -60, -100 hPa) and the pressure plate method (water potentials of -300, -1000, -3000 and -13000 hPa). Water potential was stepwise decreased in both apparatuses and the mass of the cylinders noted before a new water potential was applied. Each pressure step was equilibrated for about 7 days before the next step was applied. Finally, the cylinders were dried at 105°C and the dry weight was recorded. The difference between dry weight and weight after each pressure step divided by the cylinder volume resulted in the soil water content $\theta_{\gamma}$:

$\theta_{\gamma}=\frac{weight cylinder_{\gamma}-weight cylinder_{dry}}{1.0 \frac{g}{{cm}^{3}} * 100 cm^{3}}$ (Eq. 1)

The soil retention curve was fitted using the van Genuchten equation using Excel solver to determine model parameters:

$\theta_{\gamma}=\theta_{r}+\left( \theta_{s}-\theta_{r} \right)*{(\frac{1}{1+\left( \alpha*\gamma\right)^{n}})}^{m}$ (Eq. 2)

The model fit resulted in $\theta_{r}$= 0.01, $\theta_{s}$ = 0.49, n=1.26, $\alpha$ = 0.07 and m = 0.20 with $\theta_{\gamma}$ and $\gamma$ being water content (in vol-%) and water potential (in hPa), respectively. The quality of the model fit was estimated through an objective function that was calculated by dividing the square of measured water contents ($\theta_{\gamma\_measure}$) by the square of difference of $\theta_{\gamma\_measure}$ and model predicted water contents ($\theta_{\gamma\_estimate}$). The sum of these values was calculated, divided by the number of observations, and subtracted from 1 to calculate an objective function ($O_{f}$) equivalent to a correlation coefficient (best fit $O_{f}$ = 1):

$$O_{f}=1- \frac{\sum\frac{{(\theta_{\gamma\_measure}-\theta_{\gamma_{estimate}})}^{2}}{{\theta_{\gamma\_measure}}^{2}}}{n}$$

$O_{f}$ equaled 0.995 for our parameter fit (Figure 1).


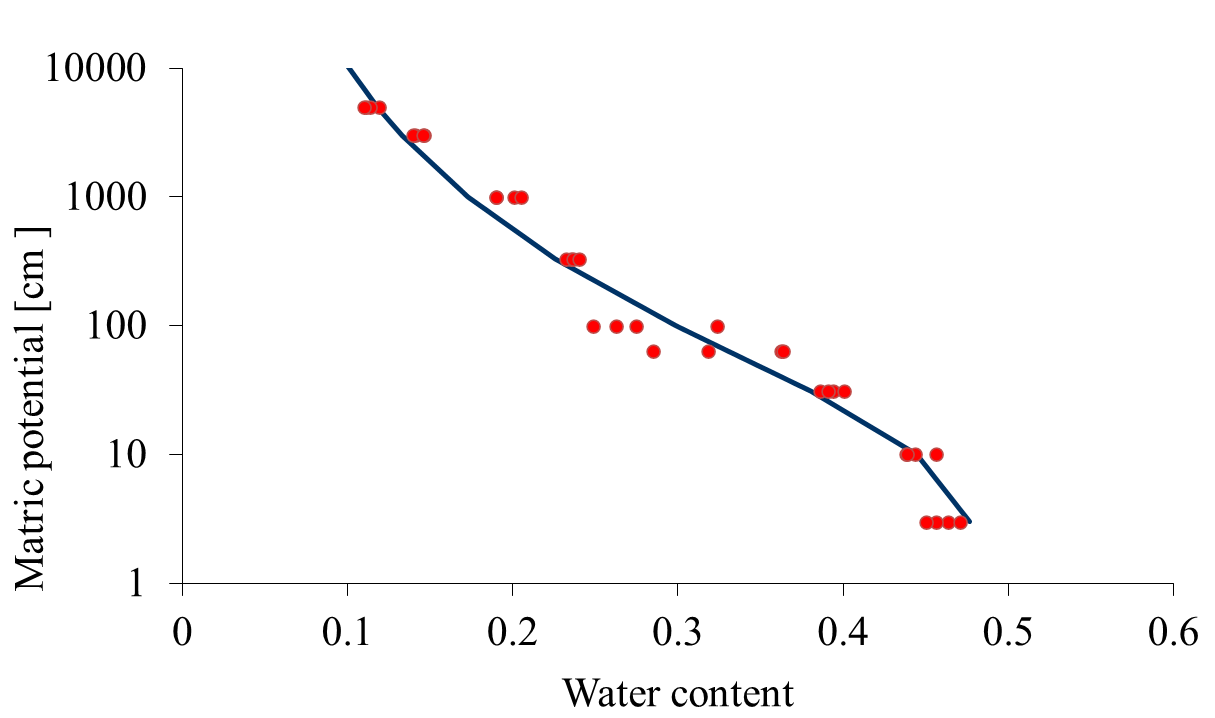


Figure 2: Measured (red dots) and fitted (blue line) parameters for soil retention curve of the soil from experiment 1.

*
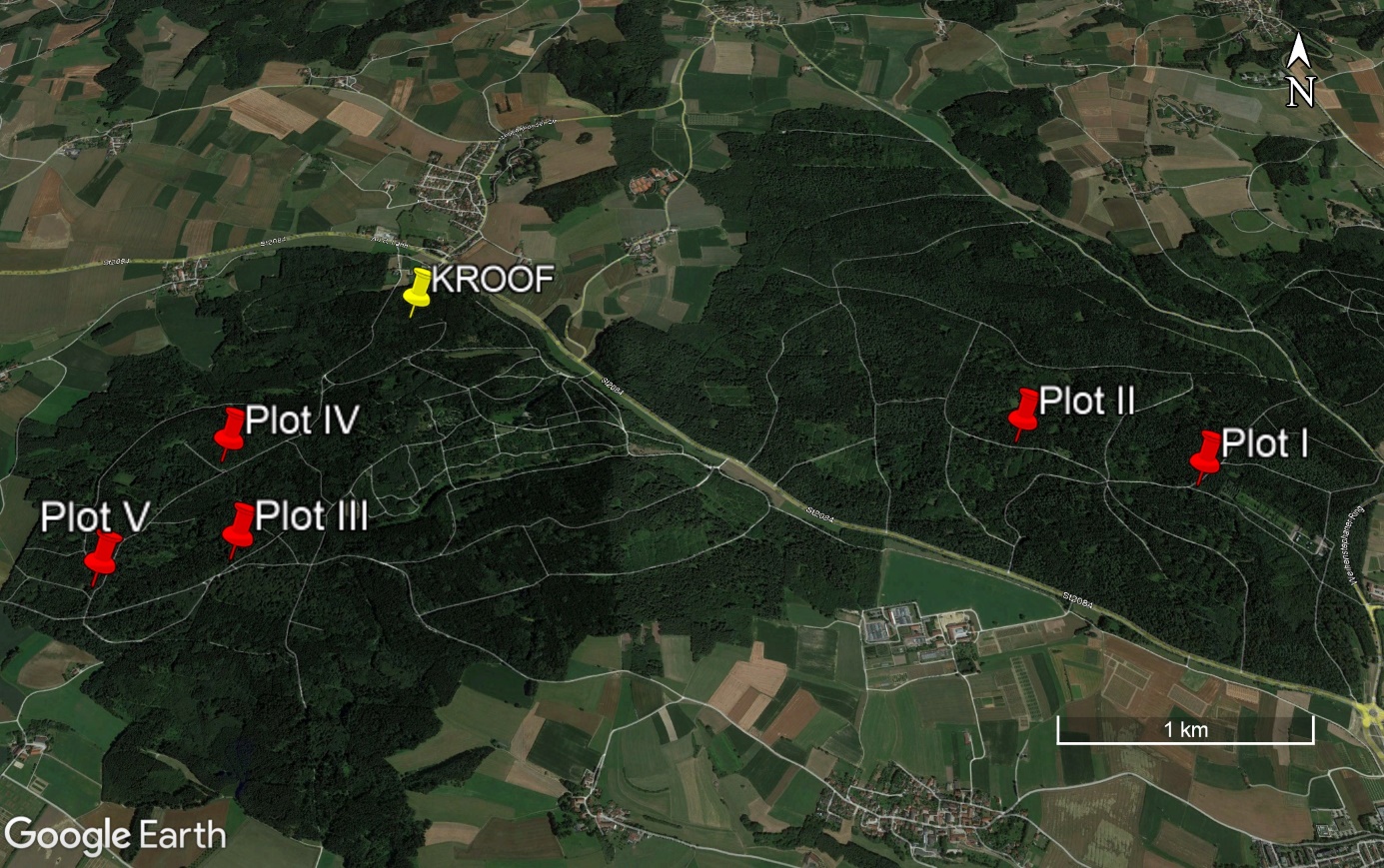
*

Figure 3: Experimental site for experiment 1 (‘KROOF’), conducted in 2017 and location of 5 experimental stands for experiment 2 in Thalhauser Forst (Plot I & II) and Kranzberger Forst (Plot III, IV and V). Picture: Google Earth Pro V.7.3.6.9750 (64-bit; Jan. 12, 2024), http://www.earth.google.com [February 16, 2024]; image from July 2016. Specific coordinates are: KROOF (48°25'9.91"N, 11°39'40.13"E), Plot I (48°24'40.29"N, 11°42'33.05"E), Plot II (48°24'47.48"N, 11°41'55.96"E), Plot III (48°24'28.71"N, 11°39'11.06"E), Plot IV (48°24'44.16"N, 11°39'4.47"E), Plot V (48°24'24.67"N, 11°38'44.40"E).

*Supplementary methods 2; determination transpirable SWC*

To estimate transpirable soil water content ($tSWC$), we used equation 6 from Klein et al. (2014):

$$\sum_{1-4} tSWC=\sum h*\left( 1-ST \right)*(SWC-ttSWC)$$

With $h$ being the height of the respective soil layers (mm), ST the stone fraction (0 in all our soil depths) and $ttSWC$ thresholds for water availability. Thresholds were taken from Grams et al. (2021) and were: 7.4, 13.5, 19.1 and 25.8 vol-% SWC for the four respective soil depths (0-7, 10-30, 30-50 and 50-70 cm). The $tSWC$ amounted to 0.6 (0-7 cm), 6.0 (10-30 cm), 6.8 (30-50 cm) and 5.8 mm (50-70 cm) or 19.2 mm across all soil depths.

Grams, T. E. E., Hesse, B. D., Gebhardt, T., Weikl, F., Rötzer, T., Kovacs, B., Hikino, K., Hafner, B. D., Brunn, M., Bauerle, T., Häberle, K. H., Pretzsch, H., & Pritsch, K. (2021). The Kroof experiment: realization and efficacy of a recurrent drought experiment plus recovery in a beech/spruce forest. *Ecosphere*, *12*(3). https://doi.org/10.1002/ecs2.3399

Klein, T., Rotenberg, E., Cohen-Hilaleh, E., Raz-Yaseef, N., Tatarinov, F., Preisler, Y., Ogée, J., Cohen, S., & Yakir, D. (2014). Quantifying transpirable soil water and its relations to tree water use dynamics in a water-limited pine forest. *Ecohydrology*, *7*(2), 409–419. https://doi.org/10.1002/eco.1360

*Supplementary methods 3; fraction of redistributed water in seedlings*

The fraction of redistributed water in seedlings at dawn ($f_{seedling dawn}$) was estimated to be a mixture between isotopic composition of the soil in 50-70 cm depth ($\delta{}^{2}{H_{soil deep}}$; assuming a similar water source as determined in experiment 1) and the isotopic composition of the seedlings in the afternoon ($\delta{}^{2}{H_{seedlings afternoon}}$; assuming that all redistributed water was used by then and the $\delta{}^{2}{H_{seedlings afternoon}}$ reflected bulk soil water at the water uptake depth):

$f_{seedling dawn}=\frac{\delta{}^{2}{H_{seedling dawn}}- \delta{}^{2}{H_{soil deep}}}{\delta{}^{2}{H_{seedlings afternoon}}- \delta{}^{2}{H_{soil deep}}}$ (Eq. 3)

Where $\delta{}^{2}{H_{seedling dawn}}$ reflects isotopic composition in seedlings’ roots at dawn.
